# Neurochemical Characterization of Brainstem Pro-Opiomelanocortin Cells

**DOI:** 10.1210/endocr/bqaa032

**Published:** 2020-03-13

**Authors:** Teodora Georgescu, David Lyons, Barbora Doslikova, Ana Paula Garcia, Oliver Marston, Luke K Burke, Raffaella Chianese, Brian Y H Lam, Giles S H Yeo, Justin J Rochford, Alastair S Garfield, Lora K Heisler

**Affiliations:** 1 Rowett Institute, University of Aberdeen, Foresterhill, Aberdeen, UK; 2 Department of Pharmacology, University of Cambridge, Cambridge, UK; 3 MRC Metabolic Diseases Unit, University of Cambridge Metabolic Research Laboratories, Wellcome Trust-MRC Institute of Metabolic Science, Addenbrooke’s Hospital, Cambridge, UK; 4 Centre for Neuroendocrinology & Department of Anatomy, University of Otago, Dunedin, New Zealand

**Keywords:** Pomc, leptin receptor, NTS, obesity

## Abstract

Genetic research has revealed pro-opiomelanocortin (POMC) to be a fundamental regulator of energy balance and body weight in mammals. Within the brain, POMC is primarily expressed in the arcuate nucleus of the hypothalamus (ARC), while a smaller population exists in the brainstem nucleus of the solitary tract (POMC^NTS^). We performed a neurochemical characterization of this understudied population of POMC cells using transgenic mice expressing green fluorescent protein (eGFP) under the control of a POMC promoter/enhancer (*Pomc*^*eGFP*^). Expression of endogenous *Pomc* mRNA in the nucleus of the solitary tract (NTS) *Pomc*^*eGFP*^ cells was confirmed using fluorescence-activating cell sorting (FACS) followed by quantitative PCR. In situ hybridization histochemistry of endogenous *Pomc* mRNA and immunohistochemical analysis of eGFP revealed that POMC is primarily localized within the caudal NTS. Neurochemical analysis indicated that POMC^NTS^ is not co-expressed with tyrosine hydroxylase (TH), glucagon-like peptide 1 (GLP-1), cholecystokinin (CCK), brain-derived neurotrophic factor (BDNF), nesfatin, nitric oxide synthase 1 (nNOS), seipin, or choline acetyltransferase (ChAT) cells, whereas 100% of POMC^NTS^ is co-expressed with transcription factor paired-like homeobox2b (Phox2b). We observed that 20% of POMC^NTS^ cells express receptors for adipocyte hormone leptin (LepRbs) using a *Pomc*^*eGFP*^:*LepRb*^*Cre:tdTOM*^ double-reporter line. Elevations in endogenous or exogenous leptin levels increased the in vivo activity (c-FOS) of a small subset of POMC^NTS^ cells. Using ex vivo slice electrophysiology, we observed that this effect of leptin on POMC^NTS^ cell activity is postsynaptic. These findings reveal that a subset of POMC^NTS^ cells are responsive to both changes in energy status and the adipocyte hormone leptin, findings of relevance to the neurobiology of obesity.

The rapid rise in the prevalence of obesity has emphasized the need for a greater understanding of the neurobiological mechanisms that underlie energy homeostasis and body weight. Circulating nutritional cues and neuromodulatory signals are integrated within the brain to regulate energy balance. The central melanocortin system is a critical point of convergence for many of these signals and has a fundamental role in regulating body weight. This pathway is comprised of the endogenous melanocortin agonists derived from POMC, which yield the signaling products α-melanocyte stimulating hormone (α-MSH), β-MSH, γ-MSH, adrenocorticotropic hormone (ACTH), and β-endorphin (1). Brain POMC peptides α-MSH, β-MSH, and γ-MSH compete for action with the endogenous melanocortin receptor antagonist/inverse agonist agouti-related peptide (AgRP) at melanocortin-3 and -4 receptors (MC3R, MC4R) (2), with MC4Rs predominantly linked to energy balance and body weight regulation (3–5). In the adult brain, POMC is expressed in the arcuate nucleus of the hypothalamus (POMC^ARC^) and the brainstem nucleus of the solitary tract (POMC^NTS^) (6, 7). However, during development, POMC is transiently expressed in at least 60 additional brain regions within cells that are not fated to be POMC (8, 9). Accordingly, studies employing *Pomc*^*CRE*^ transgenic approaches may involve recombination in off-target sites within the brain and peripheral tissues and should be interpreted accordingly.

Brain infusion of α-MSH or synthetic MC4R agonists reduce food intake and body weight, increase energy expenditure, and improve glucose homeostasis (10–12). Complementing these pharmacological studies, loss-of-function mutations of *Pomc* or *Mc4r*, or over-expression of *Agrp*, promote hyperphagia, obesity, and insulin resistance in multiple species, including zebrafish, mice, dogs, and man, illustrating the strong translational nature of this system (13–23). Furthermore, genetic haploinsufficiency of *Pomc/POMC* in both rodents and humans is associated with preferential over-consumption of dietary fat and increased risk for diet-induced obesity (DIO) (24, 25). The melanocortin system has therefore garnered substantial interest as a potential therapeutic target for obesity and the prevention of type 2 diabetes (T2D) (26, 27).

Though POMC^ARC^ neurons have been well characterized (28, 29), less attention has focused on the smaller population of POMC cells localized in the NTS. POMC^NTS^ neurons are anatomically localized to impact energy homeostasis given that (1) the NTS is a primary integrator of multiple metabolic cues (30–34), and (2) POMC^NTS^ cell activity is modulated in response to the firing of vagal afferent and ingestive-related signals (30, 31, 35). Indeed, chemogenetic activation of POMC^NTS^ neurons results in a suppression of feeding and an enhancement of short-term satiety (36). A recent report indicated that POMC^NTS^ is required for the acute anorectic effect of obesity medication 5-HT_2C_R agonist lorcaserin (37). However, what has not been established is whether other neuropeptides/neurotransmitters implicated in energy homeostasis are co-expressed with POMC^NTS^ and the direct endogenous regulators of POMC^NTS^ neuron activity.

Specifically, the NTS contains other appetite-regulating neuropeptides and neurotransmitters such as cholecystokinin (CCK) (38, 39), glucagon like peptide-1 (GLP-1) (40, 41), catecholamines (39, 42), brain-derived neurotrophic factor (BDNF) (43), and nesfatin (44). Previous reports indicate that receptors (LepRbs) for the adipocyte hormone leptin are co-expressed with a subset of POMC^NTS^ neurons using a *Pomc*^*GFP*^ mouse line (43, 45), though others have not found evidence for this using a *Pomc*^*CRE*^ line (46). In both mice and humans, the absence of *LepRb/LEPRB* leads to morbid obesity, hyperphagia, insulin resistance, and decreased energy expenditure, amongst other symptoms (47–49). Illustrating that the specific subset of LepRb co-expressed with brain POMC is involved in this metabolic phenotype, selective inactivation of LepRb only in POMC cells produces a milder version of this phenotype (50, 51). However, this Cre/Lox approach does not distinguish between LepRbs expressed within POMC in the ARC or NTS. Here we aimed to clarify the distribution, neurochemical identify, and leptin responsiveness of POMC^NTS^.

## Material and Methods

### Animals

Male and female *Pomc-enhanced green florescent protein* (*Pomc*^*eGFP*^; kindly provided by Prof. Richard Simerly and Prof. Malcolm Low) (52), *LepRb-Ires-Cre::Rosa26-eGFP* (*LepRb*^*CRE:eGFP*^) (53) and *LepRb-Ires-Cre:tdTOMATO* (*LepRb*^*tdTOM*^; kindly provided by Prof. Joel Elmquist and Prof. Jeffrey Friedman) (54) mice were used. A *Pomc*^*eGFP*^:*LepRb*^*tdTOM*^ double-reporter line was produced by crossing homozygous *Pomc*^*eGFP*^ and *LepRb*^*tdTOM*^ mice. All mice were maintained on a 12-hour light, 12-hour dark cycle with *ad libitum* access to a standard laboratory chow diet and water unless indicated otherwise. All procedures performed were in accordance with the UK Animals (Scientific Procedures) Act, 1986 and with appropriate ethical approval.

### Immunofluorescent histochemistry

Mice were deeply and terminally anesthetized with pentobarbitone (50 mg/kg i.p.) and transcardially perfused with diethylpyrocarbonate (DEPC)-treated phosphate buffered saline (PBS) followed by 10% neutral buffered formalin (Sigma-Aldrich, Gillingham, UK). Brains were extracted, postfixed in 10% neutral buffered formalin for up to 6 hours, and then cryoprotected through emersion in a 20% sucrose solution in PBS for 1–2 days at 4°C. Brains were sectioned at 25 μm on a freezing sliding microtome and collected in 5 equal series.

Free-floating NTS sections were washed in PBS and incubated for 1 hour with blocking buffer (2% bovine serum albumin with 0.25% Triton X-100 in PBS). Sections were then incubated overnight at 4°C in blocking buffer with a primary antibody: chicken anti-GFP (1:500, Abcam, Cambridge, UK [RRID: AB_300798 (55)]), rabbit anti-mCherry (1:1000, Rockland Immunochemicals, Limerick, PA [RRID: AB_2614470 (56)]), rabbit antinesfatin (1:1000, Phoenix Pharmaceuticals, Burlingame, CA [RRID: AB_2737429 (57)]), mouse antityrosine hydroxylase (TH; 1:1000, Chemicon, Temecula, CA [RRID: AB_390204 (58)]), goat anticholine acetyltransferase (ChAT; 1:1000, Millipore, Billerica, MA [RRID: AB_2079751 (59)]), rabbit antinitric oxide synthase (nNOS; 1:1000, Immunostar, Hudson, WI [RRID: AB_572255 (60)]), rabbit antiseipin antibody (1:1000, Dr. D. Ito, Keio University School of Medicine, Japan (61, 62) [RRID: *AB_2819210* (63)]), and rabbit antipaired-like homeobox 2b (Phox2b; 1:1000, Abcam, Cambridge, UK [RRID: AB_10675986 (64)]). The tissue was subsequently washed in PBS and incubated for 1 hour with the corresponding secondary Alexa Fluor antibodies (1:500, ThermoFisher, Paisley, UK [RRID: AB_2340375 (65), RRID: AB_141637 (66), RRID: AB_141633 (67), RRID: AB_142540 (68)]). Sections were mounted onto microscope slides and visualized under an Axioskop II microscope (Carl Zeiss, Oberkochen, Germany) and images were taken with an AxioCam (Carl Zeiss, Oberkochen, Germany) digital camera. Sections containing the NTS were assigned a bregma level and boundaries of the NTS delineated based on neuroarchitecture and a mouse brain atlas (69). The number of *Pomc*^*eGFP*^ immunoreactive (IR) cells, neurochemically defined cells, and double-labeled cells falling within the defined regions were counted and expressed as a percentage of total POMC-eGFP-IR expressing cells in that brain slice.

### cFOS immunohistochemistry

*Pomc* ^*eGFP*^ mice were injected with mouse recombinant leptin (5 mg/kg, i.p., Merck, Whitehouse Station, NJ) or vehicle during the light cycle and food was removed. Two hours later, mice were deeply anesthetized with pentobarbitone (50 mg/kg i.p.) and transcardially perfused with DEPC-treated PBS followed by 10% neutral buffered formalin. Another cohort of *Pomc*^*eGFP*^ mice were fed ad libitum (n = 4), 12-hour fasted overnight (n = 5) or 12-hour fasted overnight followed by a 2-hour light cycle refeeding (n = 5). Mice were then injected with deep terminal anesthesia and transcardially perfused with DEPC-treated PBS followed by 10% neutral buffered formalin. Brains were extracted and tissue prepared as described above. Free-floating NTS sections were processed at room temperature. Tissue was washed in PBS and then treated for 30 minutes with 0.3% H_2_O_2_ in PBS. Tissue was then washed in PBS, blocked for 1 hour in blocking solution (0.25% Triton X-100 and 3% normal donkey serum in PBS) and incubated overnight in primary antibody rabbit anti-c-fos added to blocking solution (cFOS; 1:5000, Calbiochem, Watford, UK [RRID: AB_2106755 (70)]). The sections were next incubated in biotinylated donkey antirabbit IgG (1:1000, Jackson ImmunoResearch Laboratories, West Grove, PA [RRID: AB_2340593 (71)]) for 1 hour, followed by 2-hour incubation in an avidin–biotin complex solution (1:500, Vectastain Elite ABC Kit, Vector Laboratories, Peterborough, UK) diluted in PBS. After several washes in PBS, sections were incubated in a solution of 0.04% diaminobenzidine tetrahydrochloride (DAB) and 0.01% H_2_O_2_ in PBS. Brain tissue was then processed for GFP immunofluorescent histochemistry (IHC) and analyzed as described above. Quantitative analysis of dual-labeled cells was expressed as a percentage of the total number of POMC-eGFP-IR positive cells.

### In situ hybridization histochemistry and IHC

In situ hybridization histochemistry (ISHH) was conducted as previously described (43). Briefly, mice were injected with deep terminal anesthesia and transcardially perfused with DEPC-treated PBS followed by 10% neutral buffered formalin. Brains were extracted and tissue prepared as described above. Radiolabelled riboprobes specific to the mRNA sequences of POMC, PPG, BDNF, and CCK were used to detect gene expression. Linearized recombinant plasmids were subjected to in vitro transcription with a T7 RNA polymerase (Ambion Inc., Austin, TX) in the presence of ^35^S-labeled UTP. cRNA riboprobes were diluted to 2 × 10^7^ cpm/ml in a hybridization solution. ^35^S-labeled *Pomc* was examined within adjacent brainstem sections ranging from -6.36 to -8.24 from bregma of 2- to 6-month-old male and female wild type C57BL/6 (n = 7) and *Pomc*^*eGFP*^ (n = 7) mice. Sections mounted onto microscope slides were placed with Carestream Kodak Biomax MR single emulsion film (Sigma) in a light tight box for 5 days. Films were scanned and manually analyzed, confirming ^35^S *Pomc* within the NTS. High-resolution images were generated using an AxioCam HRc (Carl Zeiss) interface with a brightfield Axioskop II microscope (Carl Zeiss).

Following ISHH, free-floating tissue processed for dual-histochemical staining was washed in PBS before commencement of the IHC protocol. Sections were treated for 30 minutes in 0.3% H_2_O_2_ in PBS, rinsed in PBS, and blocked in 0.5% BSA/0.5% TritonX-100 in PBS for 1 hour. Sections were incubated in blocking buffer containing goat anti-GFP antibody (1:1000, Abcam, Cambridge, UK [RRID: AB_304897 (72)]) overnight. Sections were washed in PBS and a biotinylated rabbit antigoat secondary antibody (1:1000, Vector Laboratories, Peterborough, UK [RRID: AB_2336126 (73)]) in blocking buffer for 1 hour. Sections were then washed in PBS and incubated for 1 hour in VectaStain ABC reagent and chromogenic detection was conducted using DAB reagent (Vector Laboratories, Peterborough, UK). Sections were mounted onto microscope slides and air dried. Slides were dipped in photographic emulsion (Kodak, Rochester, NY) and stored at 4°C for 2 weeks before being developed in D-19 developer and fixer (Kodak). Double-labeled cells were recorded if GFP-IR-positive cell bodies contained overlying black grains that were in a quantity greater than 3 times the background and conformed to the shape of the GFP-IR cell bodies.

### Isolation of NTS POMC-GFP cells with FACS

Four-week-old male and female ad libitum–fed *Pomc*^*eGFP*^ mice (n = 6) were sacrificed between 9:00 and 10:00 am. The NTS was microdissected into ice cold dissociation buffer consisting of 0.36% glucose (Sigma, Gillingham, UK), 2 × 10^-4^% phenol red solution (Sigma), and 1 mM HEPES buffer (Sigma) in HBSS solution (Invitrogen, Paisley, UK). 10X Ky/Mg solution consisting of 0.19% kynurenic acid (Sigma), 5 × 10^-4^% phenol red solution (Sigma), 5 mM HEPES buffer (Sigma), and 0.1M MgCl_2_ (Sigma), pH 7.5, was added before use. Dissociation media was replaced with 1 ml prewarmed 20 U/ml papain solution consisting of 20X L-cysteine solution (Sigma), papain solution (Sigma), and dissociation medium (containing 10X Ky/Mg) and nuclei were incubated at 37^o^C for 30 minutes. Papain solution was replaced with 1 ml prewarmed 10 mg/ml trypsin inhibitor solution (Sigma, diluted in dissociation media) and the nuclei incubated at 37^o^C for 10 minutes. Trypsin inhibitor was removed and nuclei were washed with 1 ml ice cold PBS (Sigma) with 2% FCS (P.A.A. laboratories, Ltd., Yeovil, UK). Phosphate buffered saline was replaced with 2 ml PBS with 2% FCS and the solution triturated. Samples were filtered through a 70 µm cell strainer (BD Falcon, Tewksbury, MA). Fluorescence-activating cell sorting was performed using an Influx Cell Sorter (BD Biosciences, San Jose, CA) utilizing a previous established method (31, 32). Cell gating was set according to cell size (FSC), cell granularity (SSC), FSC pulse-width for singlets, and fluorescence at 488 nm/532 nm for GFP and 647/670 nm for nuclear stain with DraQ7 (Biostatus, Shepshed, Leicester, UK). Pools of GFP-positive (GFP+) and GFP-negative (GFP-) cells were sorted into a plate containing RLT lysis buffer (Qiagen, Manchester, UK) and RNA was isolated using RNeasy Plus Micro Kit (Qiagen). Reverse transcription and whole transcriptome amplification were performed using Nugen Ovation Pico WTA system V2 following the manufacturer’s instructions. *Pomc* expression was determined by quantitative real-time PCR (qPCR) using Sybr green with primers designed specifically for *Pomc* (^Forward CTCCTGCTTCAGACCTCCAT^, ^Reverse- TTTTCAGTCAGGGGCTGTTC^), *Gfp* (^Forward- AGCCGCTACCCCGACCACAT, Reverse- CGGTTCACCAGGGTGTCGCC^). Gene expression was normalised to *Gapdh*^(Forward- TGTTCCTACCCCCAATGTGT, Reverse- GGTCCTCAGTGTAGCCCAAG^) in the same samples.

### Electrophysiology

Standard current and voltage clamp whole-cell patch clamp electrophysiology recordings were made from POMC^NTS^ cells. Specifically, *Pomc*^*eGFP*^ mice (6–10 weeks old) were injected with a terminal dose of pentobarbitone (50 mg/kg, i.p.), decapitated, brains rapidly extracted, and immediately submerged in an ice-cold solution containing 250 mM sucrose, 2.5 mM KCl, 21 mM NaHCO_3_, 1.2 mM NaH_2_PO_4_, 3 mM glucose, 4 mM MgCl_2_, and 0.1 mM CaCl_2_ bubbled with 5% CO_2_ and 95%O_2._ The brain was fixed to a vibrating microtome (Slicer HR2; Sigmann Elektronik, Hüffenhardt, Germany) and 180 μm coronal sections of the brainstem containing the NTS were prepared. The slices were then incubated for 25 minutes at 34°C in oxygenated artificial cerebral spinal fluid (aCSF) containing 125 mM NaCl, 2.5 mM KCl, 21 mM NaHCO_3_, 1.2 mM NaH_2_PO_4_, 3 mM glucose, 2 mM MgCl_2_, and 2 mM CaCl_2_. Prior to recording, slices were allowed to recover in the aCSF solution at room temperature for a minimum of 1 hour. Green fluorescent protein cells in live slices were identified using a Nikon Eclipse E6000FN microscope fitted with an LED light source (Cairn, Edinburgh, UK). During recording, unless otherwise described, slices were continuously perfused at a rate of ca. 2 ml/min with oxygenated aCSF solution at 31°C. Leptin was bath applied. Thick-walled glass pipettes (Harvard Apparatus, Holliston, MA) of 4 to 5 MΩ resistance were pulled using a Zeitz DMZ micropipette puller (Zeitz Instruments GmBH, Martinsried, Germany). Pipettes were filled with the following intracellular solution: 120 mM K-gluconate, 10 mM KCl, 0.1 mM EGTA, 10 mM HEPES, 4 mM K_2_ATP, and 1 mM Na_2_ATP at pH 7.3 (with KOH). Recordings were performed using an EPC10 amplifier and Patchmaster software (HEKA, Lambrecht/Pfalz, Germany). Where indicated, the following combination of neurotransmission inhibitors was used: 1 μM tetrodotoxin (TTX), 3 μM strychnine, 50 μM picrotoxin, 50 μM D-AP5, 10 μM CNQX. Liquid junction potential was 10 mV and not compensated. The recorded current was sampled at 10 kHz and filtered at 5 kHz unless otherwise stated. No effect was set as a change in membrane potential less than 2 mV. All compounds were dissolved in water with the exception of picrotoxin, which was dissolved in ethanol.

### Leptin ELISA

Animals were terminally anesthetized with pentobarbitone (50 mg/kg, i.p.). The blood was collected in EDTA-coated tubes (Startedt AG & Co., Numbrecht, Germany). To separate plasma, whole blood was centrifuged at 3500 rpm for 15 minutes at 4°C. Leptin concentrations in serum samples was determined using Mouse Leptin Quantikine® ELISA (R&D Systems, Abington, UK) according to the manufacturer’s protocol at room temperature. Briefly, the kit equilibrated for 30 minutes at room temperature. Assay diluent RD1W (50 μl) and sample (50 μl) were added to the 96-well microplate. Next, working leptin standards and leptin controls were added and the plate was covered for 2 hours. Each well was aspirated and washed (5x) with 400 μl wash buffer. Mouse leptin conjugate (100 μl) was added to each well and the plate was incubated for 2 hours. The washing step was repeated. Substrate solution (100 μl) was added to each well and the plate, and the plate was covered to protect it from light exposure and left for 30 minutes. Finally, the reaction was stopped by adding 100 μl of stop solution, initiating a color change from blue to yellow. The plate was mixed by gentle tapping and the absorbance was then measured within 30 minutes on an EnVision® 2104 Multilabel Plate Reader (Perkin Elmer, Waltham, MA), measuring the absorbance at 450 nm and subtracting the absorbance at 540 nm/570 nm. The serum leptin levels in each group were determined using the standard curve obtained from the leptin standards provided.

### Statistics

Data were analyzed with t-test or one-way ANOVA followed by Tukey’s post hoc tests, where appropriate. For all analyses, significance was assigned at *P* < 0.05. Data are presented as mean ± SEM.

## Results

### *Pomc mRNA and Pomc* ^*eGFP*^ distribution in the NTS

**Figure 1.**
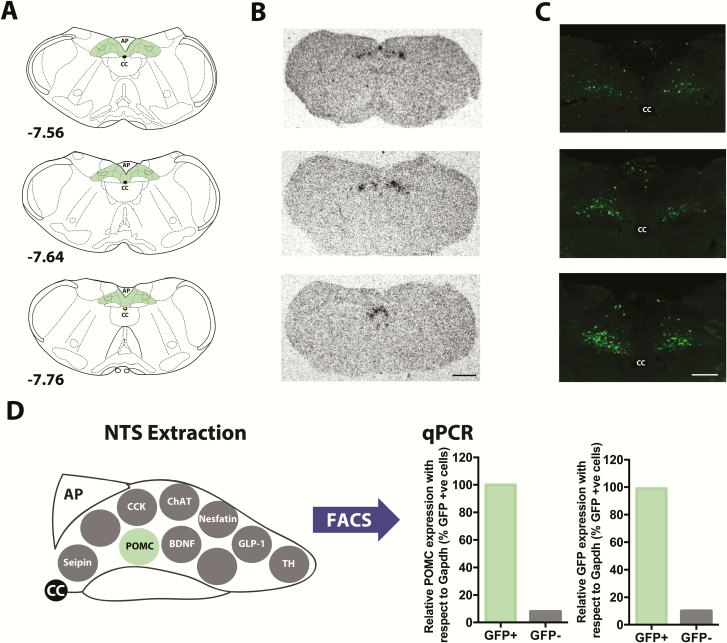
POMC^NTS^ expression and *Pomc*^*eGFP*^ validation. **A:** Adapted brain atlas (69) schematics of NTS (shaded green area) at -7.56 to -7.76 from bregma. **B:** In situ hybridization histochemistry (ISSH) with a ^35^S-labelled *Pomc* riboprobe revealed the highest level of *Pomc* mRNA (black grains) within the NTS is between -7.5 to -7.8 from bregma in adult wild-type C57BL/6 mice (n = 7). These findings were reproduced in *Pomc*^*eGFP*^ mice (n = 7). **C:** Representative photomicrographs of GFP immunofluorescence (IF) in adult male and female *Pomc*^*eGFP*^ mice (n = 20) also identified that the most abundant distribution of GFP-IR cells is between -7.5 to -7.8 from bregma**. D:** Expression of *Pomc* mRNA in GFP cells in extracted NTS of *Pomc*^*eGFP*^ mice was confirmed using fluorescence-activating cell sorting (FACS) followed by qPCR analysis. *Pomc* and *Gfp* mRNA were normalized to that of the housekeeping gene *Gapdh*. Expression of *Pomc* and *Gfp* mRNA is expressed as a percentage of that determined in GFP-positive (GFP+) cells and compared to GFP-negative (GFP-) cells. CC, central canal, scale bar B 500 μm; scale bar C 125 μm.

POMC is an essential regulator of energy homeostasis and body weight, and POMC neurons within the ARC have been well characterized (28, 29). POMC cells are also localized within the NTS. Using in situ hybridization with a ^35^S *Pomc* riboprobe, endogenous *Pomc* mRNA expression was visualized within the NTS of wild-type male and female mice on a C56BL/6 background (n = 7) and *Pomc*^*eGFP*^ mice (n = 7). ^35^S *Pomc* within the NTS was most abundant between -7.5 to -7.8 from bregma (Fig. 1A and 1B). Scattered cells were also evident at more rostral and caudal levels. POMC^NTS^ has likely received less attention compared to POMC^ARC^ because this NTS subpopulation is smaller and more difficult to visualize on a single cell level with standard histochemical techniques, as we have observed here. To overcome these technical difficulties we employed a *Pomc*^*eGFP*^ reporter mouse line to map the distribution of individual POMC cells within the brainstem. Expression of GFP protein confirmed that the majority of brainstem POMC cells sit within caudal NTS (-7.5 to -7.8 from bregma; Fig. 1C).

### NTS validation of *Pomc*^*eGFP*^ mouse line

To confirm the expression of *Pomc* mRNA within eGFP cells, the NTS was microdissected from *Pomc*^*eGFP*^ mice (n = 6), and FACS followed by *Pomc* and *Gfp* qPCR was performed (Fig. 1D). One-hundred percent of GFP-positive cells expressed *Pomc* mRNA as compared to 8% of GFP-negative cells. Similarly, 99% of GFP-positive cells expressed *Gfp* mRNA as compared to 10% of GFP-negative cells. These data provide the first NTS validation of the *Pomc*^*eGFP*^ line and verify the expression of endogenous POMC^NTS^.

### Neurochemical characterization of POMC^NTS^ neurons

**Figure 2.**
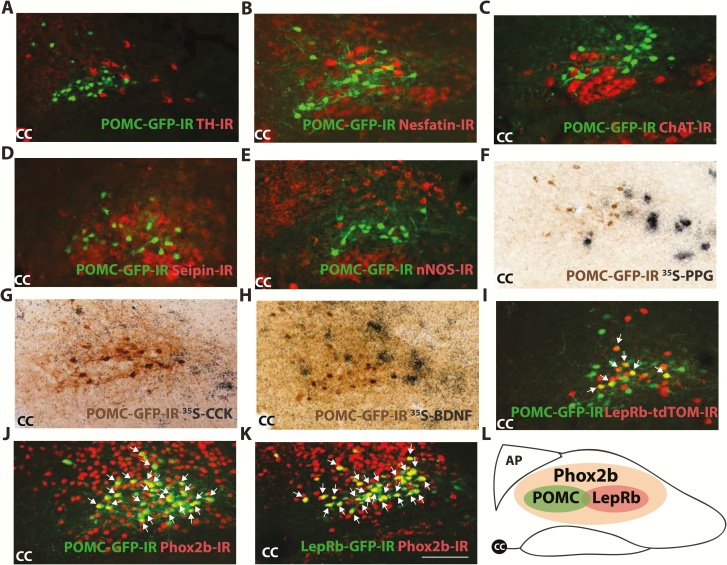
Neurochemical characterization of POMC^NTS^ neurons. Immunohistochemical analysis of adult male and female *Pomc*^*eGFP*^ mice demonstrated GFP-IR cells (green) to be negative for catecholamine cell marker tyrosine hydroxylase (TH)-IR (red) **(A)**, nesfatin-IR (red) **(B)**, acetylcholine cell marker choline acetyltransferase (ChAT)-IR (red) **(C)**, protein seipin-IR (red) **(D)** and enzyme nitric oxide synthase 1 (nNOS)-IR (red) (n = 3–5 mice per analysis) **(E)**. Dual IHC and ISHH analysis of adult male and female *Pomc*^*eGFP*^ mice demonstrated the absence of GFP-IR co-expression (brown cytoplasmic stain) with ^35^S *preproglucagon*** (***Ppg*) **(F)**, ^35^S *cholecystokinin (Cck)***(G)** or ^35^S *brain derived neurotrophic factor* (*Bdnf*) mRNA (black grains) (n = 3–5 mice per analysis) **(H)**. **I:** Double-IF analysis in adult male and female *Pomc*^*eGFP*^*:LepRb*^*Cre:tdTomato*^ mice (n = 9) revealed a subset of POMC-expressing neurons (green) were co-expressed with LepRb-expressing cells (red). **J:** In *Pomc*^*eGFP*^ mice (n = 4) and *LepRb*^*CRE:eGFP*^ mice (n = 4) **(K)**, all GFP-IR cells (green) were Phox2b-IR positive (red). **L:** Schematic illustrating overlap of POMC, Phox2b, and LepRb in the NTS. Level of NTS presented in **(A–K)**, -7.56 to -7.76 from bregma. Arrows represent dual-labeled cells. CC, central canal; scale bar, 50 μm applies to **(A–K)**.

The NTS has a regulatory role in a large number of physiological processes. As a consequence of this functional heterogeneity, this nucleus is home to numerous distinct neuronal populations. To characterize POMC^NTS^ cells, we examined the level of co-expression between *Pomc*^*eGFP*^ and other neurochemicals and receptors implicated in the regulation of energy balance. We observed that POMC^NTS^ cells do not co-localize with catecholamines epinephrine or norepinephrine using TH to visualize this subset of cells (Fig. 2A). Similarly, *Pomc*^*eGFP*^ was not co-expressed with the neuropeptide nesfatin-1 (Fig. 2B) or the neurotransmitter acetylcholine using choline acetyltransferase (ChAT; Fig. 2C). *Pomc*^*eGFP*^ was expressed in a distinct population to the protein seipin associated with lipodystrophy (Fig. 2D) and the enzyme nitric oxide synthase 1 (nNOS; Fig. 2E). Likewise, *Pomc*^*eGFP*^ was not co-localized with neuropeptides involved in the reduction of food intake, preproglucagon (PPG)/GLP-1 (Fig. 2F), CCK (Fig. 2G), or BDNF (Fig. 2H), as revealed with immunohistochemistry combined with in situ hybridization. Thus, *Pomc*^*eGFP*^ neurons appear to reside in a distinct subgroup within the NTS and do not co-express other well-characterized regulators of energy homeostasis.

We then examined potential endogenous regulators of POMC^NTS^. Some previous reports indicate that leptin administration increases a marker of leptin signal transduction (pSTAT) in *Pomc*^*eGFP*^ cells (45), thereby providing evidence of co-localization. To facilitate the visualization of POMC and LepRb cells and to quantify co-expression, we crossed *LepRb*^*tDTomato*^ mice with *Pomc*^*eGFP*^ mice to create a double reporter line (*Pomc*^*eGFP*^*:LepRb*^*tdTomato*^). We found that 20.6% ± 2.14 of POMC^NTS^ cells co-localized with LepRb^tdTomato^ (Fig. 2I). Co-localization was highest at -7.64 and -7.76 from bregma, the subregion of the NTS we found to express the majority of POMC cells (Fig. 1C). All *Pomc*^*eGFP*^ cells co-expressed Phox2b, a transcription factor associated with the formation and development of the NTS (74) (Fig. 2J). Using *LepRb*^*CRE:eGFP*^ mice to visualize LepRb^NTS^ expression, we observed that all LepRb^NTS^ cells also expressed Phox2b (Fig. 2K and 2L).

### *Pomc* ^*eGFP*^ cells increase their activity in response to food intake

**Figure 3.**
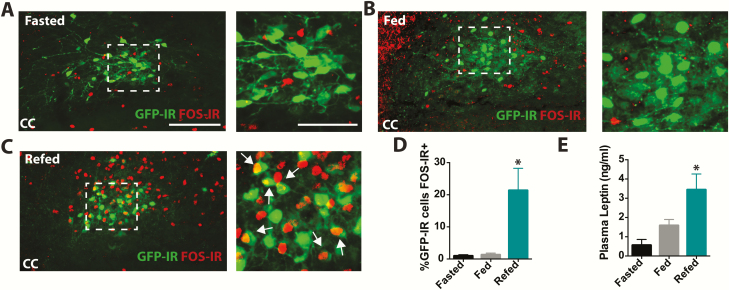
*Pomc* ^*eGFP*^ cells increase their activity in response to food intake. **A-D:***Pomc*^*eGFP*^ mice exhibit more FOS-IR in GFP-IR cells within the NTS in response refeeding compared to cells from mice in the ad libitum–fed or 12-hour fasted state (n = 4–5). **E:** Endogenous plasma leptin levels are increased following refeeding (n = 8) compared to ad libitum–fed (n = 9) or 12-hour fasted mice (n = 4) as measured by an ELISA assay. Level of NTS presented in **(A–C)**, -7.76 from bregma. Arrows represent dual-labeled cells. CC, central canal. Scale bar, 50 μm applies to **(A–C)**. Scale bar in inset, 25 μm. Data are presented as mean ± SEM, * *P* < 0.05.

Given that a subset of POMC^NTS^ cells co-express LepRb, we postulated that this subgroup should be responsive to leptin. Fasting has been shown to affect the concentrations of various circulating hormones, including reducing leptin (75). We dark cycle fasted mice, dark cycle fasted and then re-fed mice, or ad libitum fed mice and measured serum leptin and the activity of POMC^NTS^ cells using FOS-IR (Fig. 3). The NTS and blood was collected 2 to 4 hours following the onset of the light cycle. As expected, fasting significantly reduced leptin levels (0.56 ± 0.30 ng/ml, n = 4) and fasting followed by a 2-hour bout of refeeding significantly increased leptin levels (3.44 ± 0.82 ng/ml, n = 8; F_2,19_ = 4.64, *P* = 0.023; Fig. 3E). A significant difference in leptin levels between the refed group and the fed group (1.58 ± 0.32 ng/ml, n = 9) was not detected. Refeeding (n = 9) also significantly increased the number of FOS-IR-positive cells within the NTS compared to the fasted state or fed state (Fig. 3E). Significantly more NTS *Pomc*^*eGFP*^ neurons expressed FOS-IR in mice that had been fasted and then refed (21.34 ± 6.91 cells) compared to mice in the fasted (0.93 ± 0.42 cells) or fed state (1.29 ± 0.54 cells; F_2,11_ = 7.55, *P* = 0.008, n = 4–5 per group; Fig. 3A–3D). These findings suggest that a subset of POMC^NTS^ cells are responsive to nutritional state and changes in hormones such as endogenous leptin levels.

### Leptin activates a subset of POMC^NTS^ neurons

**Figure 4.**
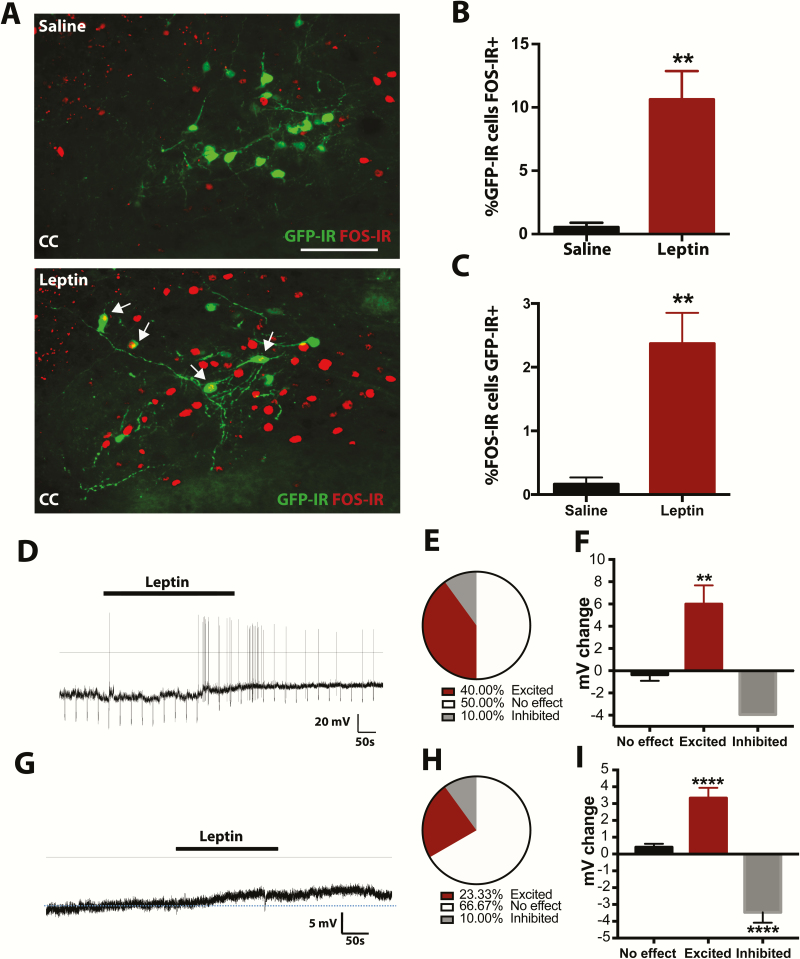
*Pomc* ^*eGFP*^ cells are responsive to changes in exogenous leptin levels. **A–C:** Leptin treatment (5 mg/kg, i.p.) in *Pomc*^*eGFP*^ mice increased FOS-IR within GFP-IR **(A–B)** and non-GFP-IR NTS cells **(C)** compared to saline treatment (n = 10). Level of NTS presented in A, -7.56 to -7.76 from bregma. **D:** Representative current clamp recording of a *Pomc*^*eGFP*^ cell. **E–F:** Bath application of leptin (100–250 nM) increased the membrane potential of 4 out of 10 *Pomc*^*eGFP*^ cells and reduced the membrane potential of 1 cell. **G:** Representative current clamp recording of a *Pomc*^*eGFP*^ cell in the presence of synaptic blockers (1 μM tetrodotoxin, 3 μM strychnine, 50 μM picrotoxin, 50 μM D-AP5, 10 μM CNQX). **H–I:** Bath application of leptin (250–500 nM) depolarised and increased the membrane potential of 7 of 30 *Pomc*^*eGFP*^ cells and inhibitory effects were observed in 3 cells. Arrows represent dual-labeled cells. CC, central canal; scale bar A, 50 μm. Data are presented as mean ± SEM. ** *P* < 0.01, **** *P* < 0.0001.

To gain further insight into the physiological role of the LepRb expression on POMC^NTS^ cell activity, we examined the effect of peripheral administration of leptin. Consistent with the fluctuations of endogenous leptin described above, we observed that leptin (5 mg/kg, i.p.) increased FOS-IR within the NTS compared to saline treatment (Fig. 4A). We examined the effect specifically in POMC^NTS^ cells and found that leptin increased FOS-IR within POMC-eGFP-IR cells compared with saline (t(8) = 4.44, *P* = 0.001, n = 5 per group; Fig. 4B). However, leptin also increased FOS-IR in chemically undefined NTS cell populations. Only 2.37% of FOS-IR-positive cells were POMC-eGFP-IR (Fig. 4C).

To examine the leptin-associated activation of POMC-eGFP-IR cells further, we measured the effect of leptin on the electrical activity of POMC^NTS^ cells ex vivo. Bath application of leptin (100–250 nM) increased the firing rate of 40% of POMC neurons (6.00 ± 1.67 mV vs. -0.36 ± 0.55 mV, t(8) = 3.98, *P* = 0.005, n = 4/10 cells; Fig. 4D–4F). This effect was postsynaptic, as the depolarization persisted in the presence of synaptic blockers (3.33 ± 0.61 mV vs. -0.41 ± 0.20 mV, F_2,27_ = 41.61, *P* < 0.0001, n = 7/30 cells; Fig. 4G–4I). The in vivo and ex vivo data highlight that a subset of *Pomc*^*eGFP*^ neurons in the NTS respond to leptin.

## Discussion

Obesity has emerged as a key challenge to human health, making it essential that the neurobiology underpinning energy homeostasis is clarified to foster the identification of new strategies to prevent and treat this condition. Genetic analysis has revealed that a functioning melanocortin system is necessary for healthy body weight in multiple species. Specifically, genetic disruption of *POMC/Pomc* and *MC4R/Mc4r* promotes severe hyperphagia and obesity (76). Within the adult brain POMC is predominantly expressed within the ARC, and the function of POMC^ARC^ has been extensively characterized. Here we investigated the smaller and less well-studied population within the NTS.

### Distribution of POMC^NTS^

Low levels of endogenous *Pomc* expression within the NTS have hampered the study of this subpopulation. To overcome these technical restrictions, we utilized a reporter mouse line that allowed us to map, quantify, and characterize these cells. Here we provide an instrumental validation of the expression of endogenous *Pomc* mRNA in eGFP cells in the *Pomc*^*eGFP*^ mouse line. We report that POMC^NTS^ cells are primarily localized within the commissural NTS at the level of the AP. Though other energy balance modulating neurons are present within the caudal DVC, we observed a lack of co-localisation with CCK, GLP-1, TH, BDNF, nesfatin, nNOS, seipin, or ChAT-containing cells. POMC^NTS^ cells are activated by visceral afferents and chemogenetic activation of POMC^NTS^ decreases acute food intake and produces opioid analgesia and bradycardia (30, 36, 77). This suggests that POMC^NTS^ cells may constitute a distinct hub within the hindbrain for the regulation of food intake via peptide product α-MSH and for the induction of analgesia via peptide product β-endorphin. The melanocortin system also appears to play a role in blood pressure and heart rate. Compared to equally obese control subjects, the prevalence of hypertension in MC4R-deficient people was significantly lower, and MC4R-deficient patients exhibited lower increases in heart rate upon waking (78). Though lacking direct co-expression, POMC^NTS^ cells may form local interactions with neighboring DVC TH, GLP-1, CCK, BDNF, nesfatin, nNOS, seipin, and/or ChAT cells to impact homeostasis, analgesia, and/or blood pressure.

In support of system connectivity, CCK increases the firing rate of POMC^NTS^ cells (30). Though CCK is widely expressed within the CNS, recent reports support a functional role of the subpopulation of CCK-positive neurons within the NTS in feeding behavior and body weight regulation. Specifically, chemogenetic or optogenetic activation of CCK^NTS^ neurons potently reduces food intake and body weight (38, 39). This effect appears to be mediated via transmitter release in the paraventricular nucleus of the hypothalamus (PVH) and parabrachial nucleus (PBN) given that selective optogenetic activation of CCK^NTS^ axon terminals within these regions reduces feeding (38, 39). A subset of CCK^NTS^ cells co-express PPG/GLP-1 (43). Like CCK^NTS^, chemogenetic activation of PPG/GLP-1^NTS^ cells also suppresses acute feeding (41).

Nesfatin-1 is a peptide and neuropeptide first identified in 2006 and observed to reduce food intake (79). Nesfatin^NTS^ cells are responsive to CCK-8 and gastric distention (80–82). It is therefore possible that CCK^NTS^ not only impacts feeding via a PVH and PBN circuit, but also via a local interaction with nesfatin and POMC. Providing support for potential cardiovascular function, NTS microinfusion of nesfatin-1 increases blood pressure and heart rate (80). A subset of Nesfatin^NTS^ cells co-express GABA and the majority of the dorsal motor nucleus of the vagus (DMX) nesfatin cells co-express ChAT. Nesfatin^DMX^ cells innervate the stomach (80).

One of the best described cell types within the NTS modulating feeding behavior are those expressing the catecholamines. Similar to our findings, Fan and colleagues reported no overlap between the expression of POMC and TH in the NTS (31). Like POMC^NTS^ cells, vagal afferents also directly innervate TH^NTS^ cells (83) and similar to nestfatin^NTS^, TH^NTS^ are responsive to gastric distension (84). Direct chemogenetic or optogenetic activation of noradrenergic dopamine β-hydroxylase (DBH)-expressing NTS neurons also decreases food intake and body weight (39). Supporting a cardiovascular function, DBH conjugated to the neurotoxin saporin, but not the neurotoxin 6-hydroxydopamine (6-OHDA) infused into the NTS, causes myocardial lesions and in some cases sudden death (85, 86).

As observed in *POMC* deficiency, genetic alterations in *BDNF* in humans is linked to elevated food intake and obesity (87, 88). BDNF function is best characterized within the ventromedial hypothalamus (VMH) (89). BDNF signaling within the NTS is essential to life, as the knockout of its receptor TrkB within the NTS is lethal (90). Heterozygous mice with partial knockdown are hyperphagic but display a normal body weight (90). Additional research is required to define a function for BDNF^NTS^.

Despite being known primarily for its role in adipocyte differentiation, the protein seipin is expressed within the NTS; however, its function within this region has yet to be fully explored (62). nNOS plays a role in a variety of processes, including blood pressure, energy homeostasis, and synaptic plasticity. Viral knockdown of nNOS^NTS^ in rats inhibited sympathetically mediated baroreflex (91), though food intake and body weight in these rats was not reported and remains to be investigated. Thus, the NTS harbors a variety of factors involved in homeostatic regulation and future study is warranted to unpick potential local interactions and their functional implications.

Though POMC^NTS^ cells do not co-express CCK, GLP-1, TH, BDNF, nesfatin, seipin, or nNOS, 100% of POMC^NTS^ co-express Phox2b. Phox2b is a transcription factor that is required for the embryonic development of the autonomic nervous system. The *Phox2b*^*CRE*^ line is frequently used as a broad NTS histochemical marker or to genetically alter cells within the NTS. Functionally, subsets of Phox2b cells have been reported to be involved in central excitatory relays of the sympathetic baroreflex and transmit peripheral chemoreceptor information to the retrotrapezoid nucleus (92, 93). Moreover, a recent study revealed that a subset of Phox2b^NTS^ neurons act as central respiratory chemoreceptors (94).

### Modulation of POMC^NTS^

In line with previous findings (31), we observed that the activity (as detected by FOS-IR) of a small subset of POMC-eGFP^NTS^ cells is activated by a feeding bout. In the present report, we employed an acute fast followed by 2 hours of refeeding. In the earlier report, *Pomc*^*eGFP*^ mice were on a restricted feeding schedule of 5 hours per day (31). The proportion of POMC^NTS^ cells activated following refeeding was greater than that observed in response to leptin treatment, suggesting that other homeostatic factors modulate the activity of POMC^NTS^ cells. We found that POMC^NTS^ cells expressing LepRbs were primarily concentrated -7.56 to -7.92 from bregma, an area where POMC neurons have been shown to respond to dietary amino acids (95, 96). A study by Grill and colleagues provides support that LepRbs within the NTS are required for appropriate energy homeostasis (97). Specifically, they observed that viral knockdown of LepRb within the NTS increased food intake, body weight, and adiposity in rats. *Phox2b*^*CRE*^*:LepRb*^*flox’d*^ mice were hyperphagic, displayed increased food intake after fasting, and gained weight at a faster rate than wild-type controls. *Phox2b*^*CRE*^*:LepRb*^*flox’d*^ mice also exhibited an increased metabolic rate independent of changes in locomotor activity and normal glucose homeostasis (98). These data suggest that leptin signaling within the NTS is required for normal feeding and body weight in rodents and that a subset of POMC^NTS^ cells are regulated by leptin.

In line with other earlier work, we observed increased FOS-IR in a subpopulation of POMC^NTS^ cells following acute leptin treatment (45, 99). However, our results do not coincide with similar experiments utilising a *Pomc*^*CRE*^*-*driven reporter line (46). As the *Pomc*^*eGFP*^ and the *Pomc*^*CRE*^ reporter lines appear to label nonoverlapping neuronal populations within the NTS (8), it is likely that the cells characterized here and in references 45 and 99 represent a different population to those studied in reference 46. To further investigate the mechanism by which POMC^NTS^ neurons respond to leptin, we employed whole cell patch clamp electrophysiology. Leptin’s effects on neuronal excitability in the rat brainstem are varied and appear to encompass cells that are inhibited, excited, or nonresponsive (100, 101). Approximately half of the unidentified neurons described in these studies became hyperpolarized upon leptin application, while a smaller proportion (13%) showed an increase in membrane potential (100, 101). We provide some of the first insights into the effect of leptin on *Pomc*^*eGFP*^ cells. We found that leptin increased the firing rate of approximately a quarter of *Pomc*^*eGFP*^ cells, a pattern similar to the proportion of POMC^NTS^ FOS-IR-positive cells. As the membrane potential increase endured in the presence of synaptic blockers, we conclude that POMC^NTS^ cells possess the required cellular machinery to respond directly to circulating levels of this hormone. However, the effects observed were small and warrant further investigation with different concentrations of leptin and different electrophysiological techniques to further clarify both the network and direct effects of leptin on *Pomc*^*eGFP*^ cell activity. TRPC channels are responsible for the activation of POMC^ARC^ neurons by leptin (102). The precise electrophysiological mechanism and the current responsible for POMC^NTS^ effects also remain to be elucidated.

We recently reported that approximately 40% of POMC^NTS^ cells co-express the 2c receptor subtype for the neurotransmitter serotonin (5-HT_2C_R) (37). Moreover, we observed that POMC^NTS^ is essential for the appetite suppressive effects of the 5-HT_2C_R obesity medication lorcaserin (37). The findings reported here add to the profile of POMC^NTS^ and its potential physiological role. Specifically, we dissect the interplay of 2 of the most essential players in body weight regulation, the melanocortin and leptin systems, within the key homeostatic brain region the NTS. We reveal that POMC^NTS^ cells represent a segregated class of leptin responsive neurons that do not co-localize other established NTS metabolic regulators. These findings provide new insights into the neuroendocrinology of appetite and have implications for the neurobiology of obesity.

## Notes

***Financial Support:*** Work was supported by the Wellcome Trust (WT081713, WT098012 and 204815/Z/16/Z to LKH; 093566/Z/10/A to LKH/LKB), the Biotechnology and Biological Sciences Research Council (BB/K001418/1, BB/NO17838/1 to LKH), and the Medical Research Council (MRC; MC/PC/15077 to LKH). The Genomics and Transcriptomics Core facility utilized was supported by the MRC (MRC_MC_UU_12012/5) and Wellcome Trust (100574/Z/12/Z).

## Additional Information

***Disclosure Summary:*** The authors have nothing to disclose.
